# High Concentration of Protein Oxidation Biomarker O-Tyr/Phe Predicts Better Outcome in Childhood Bacterial Meningitis

**DOI:** 10.3390/antiox12030621

**Published:** 2023-03-02

**Authors:** Emilie Rugemalira, Irmeli Roine, Julia Kuligowski, Ángel Sánchez-Illana, José David Piñeiro-Ramos, Sture Andersson, Manuel Leite Cruzeiro, Máximo Vento, Tuula Pelkonen

**Affiliations:** 1 Children’s Hospital, Pediatric Research Center, Helsinki University Hospital, Stenbäckinkatu 9, 00029 Helsinki, Finland; 2 Faculty of Medicine, University of Helsinki, Yliopistonkatu 4, 00014 Helsinki, Finland; 3 Faculty of Medicine, University Diego Portales, Avenida Manuel Rodríguez Sur 343, Santiago 8370109, Chile; 4 Health Research Institute La Fe (IISLAFE), Avenida Fernando Abril Martorell 106, 46026 Valencia, Spain; 5 Department of Analytical Chemistry, University of Valencia, Dr. Moliner 50, 46100 Burjassot, Spain; 6 Hospital Pediátrico David Bernardino, Rua Amilcar Cabral, Luanda, Angola; 7 Division of Neonatology, University and Polytechnic Hospital La Fe (HULAFE), Avenida Fernando Abril Martorell 106, 46026 Valencia, Spain

**Keywords:** oxidative stress, protein oxidation, biomarker, ortho-tyrosine, children

## Abstract

Neuronal damage in bacterial meningitis (BM) partly stems from the host´s inflammatory response and induced oxidative stress (OS). We studied the association of cerebrospinal fluid (CSF) biomarkers indicating oxidative damage to proteins with course of illness and outcome in childhood BM in Angola. Ortho-tyrosine/phenylalanine (o-Tyr/Phe), 3-chlorotyrosine/para-tyrosine (3Cl-Tyr/p-Tyr), and 3-nitrotyrosine/para-tyrosine (3NO_2_-Tyr/p-Tyr) concentration ratios were measured in 79 BM admission CSF samples, employing liquid chromatography coupled to tandem mass spectrometry. Besides death, disease outcomes were registered on Day 7 of treatment and one month after discharge (control visit). The outcome was graded according to the modified Glasgow Outcome Scale (GOS), which considers neurological and audiological sequelae. Children with a o-Tyr/Phe ratio below the median were more likely to present focal convulsions and secondary fever during recovery and suboptimal outcome (GOS < 5) on Day 7 and at control visit (odds ratio (OR) 2.85; 95% CI 1.14–7.14 and OR 5.23; 95% CI 1.66–16.52, respectively). Their most common sequela was ataxia on Day 7 and at control visit (OR 8.55; 95% CI 2.27–32.22 and OR 5.83; 95% CI 1.12–30.4, respectively). The association of a higher admission CSF o-Tyr/Phe ratio with a better course and outcome for pediatric BM points to a beneficial effect of OS.

## 1. Introduction

One of the key causes of neuronal damage in bacterial meningitis (BM) is bacterial toxin release and the host’s inflammatory response where oxidative/nitrosative stress plays a major pathophysiological role [1,2]. The bacterial stimulus in the subarachnoid space leads to a complex immunological cascade where cytokines and other inflammatory mediators are produced, consequently attracting polymorphonuclear leukocytes (PMNs) to the site. Amid the earliest inflammatory mediators released by PMN are large amounts of reactive oxygen and nitrogen species (ROS, RNS) that act as antimicrobials and generate oxidative/nitrosative stress, characterized as an imbalance of oxidant production and antioxidant defenses. This inflammatory reaction is beneficial for the defense against the infection but simultaneously deleterious as the pro-oxidant status can lead to structural or functional oxidative/nitrosative damage to proteins, DNA, and lipids. This can be assessed by the detection of specific biomarkers in the cerebrospinal fluid (CSF) [3,4].

The amino acid phenylalanine (Phe) can be used as a sentinel for protein damage because it has only one physiological end-product, para-tyrosine (p-Tyr), into which it is enzymatically oxidized by the action of phenylalanine hydroxylase. Under conditions of oxidative stress, the hydroxyl radical (∙OH) oxidizes the benzyl ring of Phe into the abnormal tyrosine isomers ortho-tyrosine (o-Tyr) and meta-tyrosine (m-Tyr), whereas p-Tyr is oxidized by peroxynitrite (ONOO−) into 3-nitrotyrosine (3NO_2_-Tyr) or by hypochlorous acid (HClO) into 3-chlorotyrosine (3Cl-Tyr). O-Tyr, m-Tyr, 3NO_2_-Tyr and 3Cl-Tyr are considered reliable biomarkers of oxidative protein damage [5]. The synthesis of the biomarkers is represented as figures in another context [6].

In addition to ROS, matrix metalloproteinases (MMPs) and the tissue inhibitor of metalloproteinases (TIMP)-1 contribute to the pathogenesis of BM brain damage. MMPs are proteolytic enzymes that take part in tissue destruction and remodeling, and act as modulators of inflammation [7]. Previous studies suggest a critical role for MMP-8 and MMP-9 as effectors of blood–brain barrier (BBB) damage [8,9] and, furthermore, oxidative stress as a trigger for the activation of MMPs [10]. 

If persistent, the inflammatory state in BM subsequently leads to decreased cerebral perfusion, cerebral oedema, raised intracranial pressure, metabolic disturbances, and vasculitis, all contributing to neuronal injury and ischemia [11]. If not fatal, there is roughly a 50% risk of short- and long-term neurological complications such as seizures, focal neurological deficits, hearing loss, cognitive impairment, hydrocephalus, learning disability and epilepsy [11]. 

In countries which include vaccination against *Haemophilus influenzae* type b (Hib), *Neisseria meningitidis*, and *Streptococcus pneumoniae* in their immunization programs, a decrease in the incidence of BM is observed [12]. For instance, in Angola, estimated by the Meningitis Progress Tracker by the World Health Organization, the under-five mortality rate for all cause BM has decreased from 82/100,000 in 2005 to 20/100,000 in 2019. The Hib and tridecavalent pneumococcal conjugate vaccine (PCV13) were implemented in Angola in 2006 and 2013, respectively. The widespread use of the meningococcal serogroup A (NmA) conjugate vaccine in the African meningitis belt countries started in 2010 and a continuous systematic surveillance shows that meningitis epidemics caused by NmA have not been reported for more than 10 years [13]. However, the BM case fatality rate has not changed significantly and may still reach 50%, with the largest concentrations of mortality remaining in the sub-Saharan Africa [14]. 

To further improve BM treatment and prognosis, a better understanding is needed of the pathophysiological events that occur after activation of the host´s inflammatory pathways. There is a substantial body of work indicating high CSF levels of reactive oxygen and nitrogen species, and of antioxidants in BM patients, but the literature is very heterogeneous as determination of their concentrations has been carried out by various means [3,15,16,17,18,19,20]. Our study group has assessed the oxidative/nitrosative stress occurring in pediatric BM patients by determining biomarkers indicating oxidative/nitrosative damage to proteins in the CSF at admission to hospital [6]. As a result, BM patients were clearly distinguished from non-BM patients, with ratios of o-Tyr/Phe, 3Cl-Tyr/p-Tyr, and 3NO₂-Tyr/p-Tyr being 570, 20, and 4.5 times as high, respectively.

Recently, BM research has focused on interventions aimed at reducing the inflammation. For now, dexamethasone is the only adjunctive therapy recommended by most guidelines in developed countries in community-acquired BM and mainly in adults [21]. Antioxidant treatments to counteract the reactive oxygen and nitrogen species released have been studied as adjuvant treatment, among other treatments, in experimental BM models, but not in patients [22,23]. Only a limited number of human studies are available on oxidative stress in relation to outcome [18,19,20,24]. Here, we aimed to study the relation of CSF protein oxidation biomarker levels to the course and outcomes of BM.

## 2. Materials and Methods

### 2.1. Patient Data

In this sequential pediatric study, the initial patient data were collected from a prospective single-center study carried out in the Pediatric Hospital of Luanda, Angola (2005–2008, ISRCTN62824827), that examined the effect of continuous β-lactam infusion and oral paracetamol on the outcome of BM without ultimately improving it (n = 723) [25]. 

CSF samples of study participants were taken on admission to hospital. BM was defined as confirmed when a child displayed signs and symptoms of BM and had either produced a positive CSF culture, CSF polymerase chain reaction result, a positive Gram-stain result, or a positive latex-agglutination test. 

After primary CSF analysis, and whenever surplus was available, the samples were stored at −80 °C until further processing. Subsequent measurements could be performed on 79 CSF samples from confirmed BM patients. The details of Phe, p-Tyr, o-Tyr, 3Cl-Tyr, and 3NO₂-Tyr analysis were recovered employing liquid chromatography coupled to tandem mass spectrometry (LC-MS/MS) and the results, as median concentrations, are illustrated in the first part of this sequential study [6]. The MMP-8 concentration (detection limit 0.08 ng/mL) was determined by a time-resolved immunofluorometric assay (IFMA) and the MMP-9, along with TIMP-1 concentrations, by an enzyme-linked immunosorbent assay; both methods were described previously [26,27].

Data on the patient history, admission status, and the course and outcome of illness were obtained. Besides death, disease outcome by predetermined criteria was registered on Day 7 of treatment and one month after discharge. First, the category “severe neurological sequelae” included blindness, quadriplegia, hydrocephalus requiring a shunt, and/or severe psychomotor retardation (not sitting/walking, speaking, or establishing contact, or requiring institutionalization), whereas “any neurological sequelae” covered these along with hemiparesis or monoparesis, ataxia (slight, moderate, or severe), and/or moderate psychomotor impairment. Second, hearing was registered with brainstem auditory evoked potentials using threshold levels of 40 dB, 60 dB, and 80 dB. Hearing was deemed impaired if the threshold stimulus at 40 dB remained undetected. The cutoff levels for moderate and severe hearing impairment were 60 dB and 80 dB, respectively. Finally, the outcome was graded according to the modified Glasgow Outcome Scale (GOS) in which score 1 indicates death; score 2, a vegetative state; score 3, severe disability (severe neurological sequelae and/or the better ear’s threshold above 80 dB); score 4, moderate disability (hemiparesis, moderate psychomotor retardation, or a hearing threshold for the better ear above 60 dB and no higher than 80 dB); and score 5, mild or no disability (monoparesis, ataxia, or a better-ear hearing threshold between 40 dB and 60 dB). 

### 2.2. Statistical Analysis

The biomarker concentrations of o-Tyr, 3Cl-Tyr, and 3NO₂-Tyr were normalized with their corresponding precursors Phe and p-Tyr. Values below the limit of quantification (LOQ) were replaced by their corresponding 0.5 × LOQ. The biomarker concentration associations with patient baseline characteristics, course of illness and outcome measures were determined using Spearman’s rank correlation and the Mann–Whitney U-test, as appropriate. To evaluate the prognostic value of these associations, we then calculated the odds ratios (ORs) for an above- or below-median biomarker level with 95% confidence intervals (CIs). The statistical analysis was carried out with IBM SPSS Statistics 27.

### 2.3. Ethics

The study was conducted in accordance with the Declaration of Helsinki. The Luanda Children’s Hospital ethics committee approved the study in 2005, including the amendments for the current study. In the original studies, the participants were enrolled only after obtaining the consent of the legal guardian.

## 3. Results

### 3.1. Study Group

The median age of the patients was 12 months (IQR 7–42). Their median Glasgow Coma Score (GCS) at admission was 11 (IQR 7–14). A total of 60% (47/79) of the children had had seizures prior to admission and 43% (34/79) at admission. By Day 7, after the start of treatment, 35% (26/79) of the children had died, 57% (29/51) manifested neurological sequelae, 8% (4/48) manifested severe neurological sequelae, and 36% (16/45) had hearing impairment. The patient characteristics, CSF and blood sample results, and causative agents are illustrated in Table 1.

### 3.2. The Associations between o-Tyr/Phe Ratio and Admission Findings

The median o-Tyr/Phe ratio was 0.002 (IQR 0.001–0.013). A lower o-Tyr/Phe ratio (Supplemental digital content, Tables S1 and S2) distinguished boys from girls (*p* = 0.020) and characterized patients whose preadmission symptoms had lasted more than 3 days vs. less than 3 days (*p* = 0.047), and those with a history of preadmission/admission seizures vs. those with no seizures (*p* = 0.013 and *p* = 0.001, respectively).

A higher o-Tyr/Phe ratio correlated with a higher CSF leukocyte count (rho 0.384, *p* = 0.00047) and a higher admission axillary temperature (rho 0.229, *p* = 0.004). A lower o-Tyr/Phe ratio correlated with a higher CSF TIMP-1 level (rho −0.503, *p* < 0.0001) and a higher 3NO_2_/p-Tyr ratio (rho −0.351, *p* = 0.002). 

Expressing the results in ORs (Table 2), the patients with an admission CSF o-Tyr/Phe ratio below the median were 4.35 (95% CI 1.67–11.33) times more likely to present with seizures on admission. They also had increased odds for showing a below-median CSF leukocyte count, an above-median TIMP-1 level, and an above-median 3NO_2_/p-Tyr ratio (OR 3.71; 95% CI 1.47–9.42, OR 8.70; 95% CI 3.16–23.99 and OR 5.25; 95% CI 2.01–13.70), respectively. 

### 3.3. The Associations of o-Tyr/Phe Ratio with Course of Illness

The o-Tyr/Phe ratio was lower (Supplemental digital content, Table S3) in patients with seizures during hospital stay or focal seizures at any time during illness and in patients who had secondary fever subsequent to Day 7 after the start of treatment, compared with patients who did not present with seizures during hospital stay, have focal seizures during illness, or have secondary fever (*p* = 0.022, *p* = 0.039, and *p* = 0.009, respectively). 

Expressed in ORs (Table 2), the children whose admission CSF o-Tyr/Phe ratio was below the median had an OR of 3.34 (95% CI 1.08–10.39) for having secondary fever and an OR of 2.86 (95% CI 1.14–7.16) for having focal seizures during illness. 

### 3.4. The Associations of o-Tyr/Phe Ratio with Outcome

Although the median o-Tyr/Phe ratio was lower in patients who died within 24 h or by Day 7 after beginning treatment, the differences were not significant (*p* = 0.756 and *p* = 0.398, respectively: Supplemental digital content, Table S3).

However, the o-Tyr/Phe ratio was significantly lower (Supplemental digital content, Table S3) in patients with a suboptimal GOS (<5) on Day 7 of treatment and one month after discharge (*p* = 0.031 and 0.004), respectively). Likewise, the ratio was lower in patients with any neurological sequelae on Day 7 or one month after discharge (*p* = 0.0002 and *p* = 0.031, respectively) and in patients with ataxia on Day 7 of treatment or one month after discharge (*p* = 0.0002 and *p* = 0.015, respectively). Regarding hearing impairment, children showing severe hearing impairment after one month of discharge had a lower o-Tyr/Phe ratio compared with children with no, mild, or moderate hearing impairment (*p* = 0.021). 

A better outcome with a higher GOS at one month after discharge correlated with a higher o-Tyr/Phe ratio (rho 0.271, *p* = 0.036). On the other hand, a lower o-Tyr/Phe ratio correlated with greater severity of ataxia on Day 7 of treatment and one month after discharge (rho −0.557, *p* = 0.0005 and rho −0.509, *p* = 0.004, respectively).

Expressed in ORs (Table 2), the children whose o-Tyr/Phe ratio was below the median presented odds of 2.85 (95% CI 1.14–7.14) and 5.23 (95% CI 1.66–16.52) for having a suboptimal GOS on Day 7 of treatment and one month after discharge, compared with children whose o-Tyr/Phe was above the median. The suboptimal result consisted of any neurological sequalae (OR 8.55; 95% CI 2.27–32.22 and OR 8.00; 95% CI 1.51–42.45 on Day 7 of treatment and one month after discharge, respectively), principally ataxia (OR 8.55; 95% CI 2.27–32.22 and OR 5.83; 95% CI 1.12–30.40 on Day 7 of treatment and one month after discharge, respectively).

### 3.5. The Associations of 3Cl-Tyr/p-Tyr Ratio with Findings on Admission, Course, and Outcome of Disease

The only significant finding concerning the ratio 3Cl-Tyr/p-Tyr was that its lower level correlated with a longer duration of preadmission illness (rho −0.281, *p* = 0.012).

### 3.6. The Associations of 3NO_2_-Tyr/p-Tyr Ratio with Findings on Admission, Course, and Outcome of Disease

A higher 3NO_2_-Tyr/p-Tyr ratio (Supplemental digital content, Table S1) correlated with a lower systolic blood pressure (rho −0.315, *p* = 0.009) and a lower GCS (rho −0.303, *p* = 0.007) on admission. In CSF, a higher ratio correlated with a lower leukocyte count and a lower MMP-8 concentration (rho −0.264, *p* = 0.019 and rho −0.233, *p* = 0.039, respectively), but a higher glucose concentration (rho 0.319, *p* = 0.004). 

A higher 3NO_2_-Tyr/p-Tyr ratio indicated slower recovery by correlating with a longer duration with GCS under 15 (rho 0.224, *p* = 0.047).

Expressed in ORs (Table 3), the children whose 3NO_2_-Tyr/p-Tyr was above the median were 4.9 times (95% CI 1.441–16.664) more likely to have a GCS below 15 on admission, in addition to a below-median CSF leukocyte count (OR 2,976; 95% CI 1.192–7.433) and an above-median CSF glucose concentration (OR 2.56; 95% CI 1.028–6.375).

## 4. Discussion

Our findings show an association between a higher admission CSF o-Tyr/Phe ratio, reflecting a higher level of oxidative stress, and several indices attesting to a more favorable course of recovery and outcome from BM. Correspondingly, a lower admission o-Tyr/Phe ratio was associated with previously known patient admission and a course of illness characteristics that have been shown to correlate with poor outcome: low admission CSF leukocyte count, high TIMP-1, seizures, and secondary fever [28,29].

The current understanding is that the host´s excessive inflammatory reaction and further induced oxidative/nitrosative stress is highly responsible for the poor outcome in BM. Experimental studies have suggested radical scavengers and antioxidants as therapeutic options aimed at limiting BM associated neuronal injury [23]. Our results imply, on the contrary, a beneficial effect of oxidative stress on the outcome of BM, suggesting that the process is not as straightforward as assumed. For instance, an experimental study in rat pups found that treatment with the radical scavenger *α*-Phenyl-*Tert*-Butyl Nitrone (PBN) during *S. pneumoniae* meningitis attenuated cortical necrosis but increased apoptotic neuronal death in the hippocampus and further impaired learning when compared with saline-treated animals [30]. This was again in contrast with what was seen in GBS meningitis, in which PBN protected neurons from both forms of injury [31]. The authors of the former study suggested that pathogen- and host-related mechanisms that influence the redox status and transcription factors of neuronal cells could explain the protective vs. detrimental effects of the radical scavenger. 

Findings concerning the biomarker ratio 3NO_2_-Tyr/p-Tyr showed an association between a higher level of it and slower recovery, but no direct association with outcome and somewhat conflicting results with severe disease. A higher admission 3NO_2_-Tyr/p-Tyr was associated with a lower CSF leukocyte count and a below-optimal GCS under 15, measures previously shown to indicate poor outcome. Moreover, a higher 3NO_2_-Tyr/p-Tyr ratio was associated with a lower admission o-Tyr/Phe ratio. This could suggest high 3NO_2_-Tyr/p-Tyr ratio as an indicator of poor outcome. Meanwhile, inconsistent with this is the association of high 3NO_2_-Tyr/p-Tyr ratio with a high CSF glucose since the opposite, low CSF glucose, is considered a predictor of poor outcome.

No associations were seen between admission 3Cl-Tyr/p-Tyr ratio and patient characteristics, course of illness, or outcome. 

To date, there are only a few human BM studies available on oxidative/nitrosative stress in relation to outcome and even fewer involving protein oxidation/nitration. Jain et al. found that pediatric BM patients who died or had neurological sequelae had markedly elevated concentrations of cytokines (interleukin-1 beta and tumor necrosis factor alpha) and free radicals (O_2_˙^−^, H_2_O_2_) compared with BM patients with survival without sequalae. [19]. In another BM study with adults, high 3NO₂-Tyr CSF concentrations were associated with unfavorable outcome, evaluated by GOS, at discharge [18]. To our knowledge, there are no human BM studies of o-Tyr or 3Cl-Tyr levels in association with outcome available. 

We have no clear answers to explain the discrepancies between our study results and previous data, but we underline heterogenicities between them. Factors such as differences in the biomarkers measuring OS, time points of sampling, varying causative organisms, and outcome measures may play an explanatory role, at least to some degree. 

A review by Ipson et al. [32] summarizes the literature of the toxicity of abnormal tyrosine isomers to cells and tissues (in aging and in disease states such as vascular diabetic disease and cataract in the human lens) and the possible mechanism by which this may occur. In many studies, the concentrations of abnormal tyrosine isomers correlated with other indicators of disease severity. Still, the authors state that the control of oxidative stress relies not only on the antioxidant capacity of the cell but also on its ability to eliminate stable but toxic radical adducts such as m-Tyr and o-Tyr. Only when both processes fail would the diseased state become evident. More data are needed to determine whether the formation or adverse effects of the tyrosine isomers differ between tissues.

Oxidative stress is implicated as a trigger for the activation of MMPs in numerous disease pathologies [33]. An experimental pneumococcal BM study suggested the activation of MMP-9 by ROS as a target for therapeutic intervention [10]. Our results did not show an association between CSF MMP-9 or MMP-8 levels and the protein oxidation biomarkers studied, but a clear negative correlation with o-Tyr/Phe ratio and TIMP-1 was seen. We could speculate that the low CSF TIMP-1 level enables a higher inflammatory state, in addition to the high CSF leukocyte level in correlation with high o-Tyr/Phe levels, and could indicate a type of pro-inflammatory state, favoring a good outcome. 

We acknowledge study limitations which we addressed in the first part of this sequential pediatric study: the data used were from a study originally designed for other purposes. Hence, a sufficiently large CSF sample was available from only around 11% of the original series. When comparing the baseline characteristics of these two studies, we saw a difference only with pre-admission seizures: 70% (506/723) of the children had had seizures before admission in the whole study group, compared with 60% (47/79) in the current set (Supplemental material, Table S4). Access to a larger series of CSF samples, in addition to serial sampling and the study of changes in biomarker levels during treatment, together with concomitant data from other pro- and anti-inflammatory mediators, could give a better insight into the processes. Furthermore, many of the survivors were lost for follow-up, which was a challenge we anticipated in the resource-poor settings. It is possible that the most impaired children sought medical help after discharge and attended the follow-up, thus influencing the late outcome results. 

The delay in seeking medical help was rather long in our study population, and the children were in poor condition on admission to hospital. The destructive activity in BM seems to appear early, and interventions aimed at preventing brain injury may have a very limited therapeutic window to be effective [22]. Hence, more effort is needed to increase the parents’ knowledge of the signs and symptoms of BM in order to reduce the delay before admission. 

We conclude that a higher admission CSF o-Tyr/Phe ratio is associated with a better course of recovery, an outcome with fewer neurological sequelae (mainly ataxia), and a favorable GOS in pediatric BM patients. This study provides a new perspective on the host´s double-edged inflammatory reaction in terms of oxidative stress in the aim of finding new therapeutic approaches.

## Figures and Tables

**Table 1:** Patient characteristics, admission cerebrospinal fluid, and blood sample results.

Characteristics	Results
Patients	
Number of patients	79
Age in months, median (IQR)	12 (7–42)
Female sex (%)	41/79 (48%)
Weight for age, z-score below −2	19 (24%)
History of present illness	
Duration of illness in days, median (IQR)	4 (3–7)
Previous antibiotics *	30/74 (41%)
Preadmission seizures	47/79 (60%)
Findings on admission	
Seizures at admission	34/79 (43%)
Glasgow coma score, median (IQR)	11 (7–14) ^a^
Another focus of infection	19/79 (24%)
Cerebrospinal fluid	
Leukocyte count (×10⁶/L), median (IQR)	1740 (353–3515)
Glucose concentration (mg/dL), median (IQR)	16.7 (9.1–26.3) ^b^
o-Tyr/Phe, median (IQR)	0.002 (0.001−0.013)
3Cl-Tyr/p-Tyr, median (IQR)	0.007 (0.003−0.022)
3NO_2_-Tyr/p-Tyr, median (IQR)	0.001 (0.001−0.002)
MMP-8, (ng/mL), median (IQR)	481 (271–1076)
MMP-9, (ng/mL), median (IQR)	514 (205–1239)
TIMP-1 (ng/mL), median (IQR)	317 (100–1586)
Blood	
C-reactive protein on Day 1 or 2 (mg/L), median ** (IQR)	154 (81–161) ^c^
Glucose (mg/dL), median (IQR) ***	85 (62–111) ^d^
Hemoglobin Day 1 or 2 (g/dL), median (IQR)	7.5 (6–9) ^e^
Causative agent	
*Streptococcus pneumoniae*	40/79 (51%)
*Haemophilus influenzae* type b	24/79 (30%)
*Neisseria meningitidis*	11/79 (14%)

^a^ n = 78, ^b^ n = 78, ^c^ n = 34, ^d^ n = 75 and ^e^ n = 77. * Number of patients for whom data were available are shown. ** When CRP level exceeded 160mg/L, it was marked as 161mg/L. *** Lowest glucose on Day 1. The biomarker ratios of 3Cl-Tyr/p-Tyr, 3NO_2_-Tyr/p-Tyr and 3NO_2_-Tyr/p-Tyr originate from units of nmol/l.

**Table 2:** The odds ratios (OR) with 95% confidence intervals of a child with bacterial meningitis whose admission cerebrospinal fluid (CSF) o-Tyr/Phe was below the median (0.0015) in relation to patient findings on admission, course of illness, and outcome.

Characteristic	OR	95% CI	*p* Value
Admission patient findings			
Male	0.42	0.17–1.03	0.058
Seizures prior to admission	2.50	0.98–6.18	0.056
Seizures on admission	4.35	1.67–11.33	0.003
CSF leukocyte count below median	3.71	1.47–9.42	0.006
TIMP-1 level above median	8.70	3.16–23.99	<0.0001
3NO_2_-Tyr/p-Tyr above median	5.25	2.012–13.698	0.0007
Course of illness			
Secondary fever after Day 7	3.34	1.08–10.39	0.037
Seizures during hospital stay	2.32	0.89–6.03	0.085
Focal seizures during illness	2.86	1.14–7.16	0.025
Outcome			
Ataxia			
On Day 7 of treatment (n = 51)	8.55	2.27–32.22	0.002
One month after discharge (n = 31)	5.83	1.12–30.40	0.036
Moderate or severe ataxia			
On Day 7 of treatment (n = 47)	5.51	1.58–19.27	0.008
One month after discharge (n = 30)	18.67	1.90–185.4	0.012
Any neurological sequelae			
On Day 7 of treatment (n = 48)	8.55	2.27–32.22	0.002
One month after discharge	8.00	1.51–42.45	0.015
Glasgow Outcome Score below 5			
On Day 7 of treatment (n = 78)	2.85	1.14–7.14	0.026
One month after discharge (n = 60)	5.23	1.66–16.52	0.005

**Table 3:** The odds ratios (OR) with 95% confidence intervals (95% CI) of a child with bacterial meningitis whose admission cerebrospinal fluid (CSF) 3NO_2_-Tyr/p-Tyr was above the median (0.001) in relation to patient findings on admission, course of illness. and outcome.

Characteristic	OR	95% CI	*p* Value
Systolic blood pressure below median	1.071	0.150–7.642	0.945
GCS below optimal on admission *	4.90	1.441–16.664	0.011
CSF leukocyte count below median	2.976	1.192–7.433	0.020
CSF glucose above median	2.56	1.028–6.375	0.043
MMP-8 below median	1.289	0.533–3.121	0.573

* Glasgow coma scale score below 15.

## Data Availability

The data presented in this study are available on request from the corresponding author. The data are nor publicly available due to patient-related confidentiality.

## Supplementary Materials

The following supporting information can be downloaded at: https://www.mdpi.com/article/10.3390/antiox12030621/s1. Table S1: Associations between admission cerebrospinal fluid (CSF) protein oxidation biomarker ratios o-Tyr/Phe, 3Cl-Tyr/p-Tyr, and 3NO_2_-Tyr/p-Tyr and baseline patient characteristics calculated using Spearman’s Rank correlation; Table S2: Associations between admission cerebrospinal fluid protein oxidation biomarker ratios o-Tyr/Phe, 3Cl-Tyr/p-Tyr, and 3NO_2_-Tyr/p-Tyr (median) and baseline patient characteristics calculated using the Mann–Whitney U-test; Table S3: Associations between admission cerebrospinal fluid protein oxidation biomarker ratios o-Tyr-Phe, 3Cl-Tyr/p-Tyr, and 3NO_2_-Tyr/p-Tyr (median) and course of illness and patient outcome calculated using the Mann–Whitney U-test.
