# Supplementary material for: High Concentration of Protein Oxidation Biomarker O-Tyr/Phe Predicts Better Outcome in Childhood Bacterial Meningitis
Source: Antioxidants (Basel). 2023 Mar 2;12(3):621. doi: 10.3390/antiox12030621 (PMC10045379; doi:10.3390/antiox12030621)
Supplement: Supplementary file 1 [file antioxidants-12-00621-s001.zip › antioxidants-2234866-supplementary.pdf]

Table S1. Supplemental digital content. Associations between admission cerebrospinal fluid (CSF) protein oxidation biomarker ratios of o-Tyr/Phe, 3Cl-Tyr/p-Tyr, 3NO_2-Tyr/p-Tyr and baseline patient characteristics, calculated using Spearman's Rank correlation.

**Characteristic**	o-Tyr/Phe		3Cl-Tyr/p-Tyr		3NO_2-Tyr/p-Tyr	
	Rho	*p* value	Rho	*p* value	Rho	*p* value
Age, months	0.127	0.263	0.19	0.865	-0.047	0.680
Duration of preadmission illness, days	-0.215	0.057	-0.281	0.012	0.065	0.571
Axillary temperature, °C (n = 78)	0.229	0.044	0.249	0.028	-0.112	0.327
Systolic blood pressure, mmHg (n = 68)	0.032	0.798	0.099	0.423	-0.315	0.009
C-reactive protein Day 1 or 2, mg/L (n = 34)	-0.167	0.346	0.237	0.177	0.151	0.394
Glasgow Coma Score on admission (n = 78)	0.181	0.112	-0.145	0.207	-0.303	0.007
CSF leukocyte count	0.384	0.0005	0.120	0.293	-0.264	0.019
CSF glucose (n = 78)	-0.127	0.268	-0.041	0.721	0.319	0.004
MMP-8, ng/mL	0.190	0.094	0.032	0.776	-0.233	0.039
MMP-9, ng/mL (n = 78)	-0.091	0.427	0.082	0.473	-0.123	0.282
TIMP-1 ng/mL	-0.503	<0.0001	-0.097	0.394	0.131	0.249

Table S2. Supplemental digital content. Associations between admission cerebrospinal fluid protein oxidation biomarker ratios of o-Tyr/Phe, 3Cl-Tyr/p-Tyr and $3NO_2$-Tyr/p-Tyr (median) and baseline patient characteristics, calculated using Mann-Whitney U-test.

	**o-Tyr/Phe**			**3Cl-Tyr/p-Tyr**			**$3NO_2$-Tyr/p-Tyr**		
**Characteristics**	Yes	No	*p* value	Yes	No	*p* value	Yes	No	*p* value
Sex, male	1.3177×10^{-3}	9.1579×10^{-3}	0.020	5.93398×10^{-3}	7.4315×10^{-3}	0.179	1.3481×10^{-3}	1.3670×10^{-3}	0.569
Another focus of infection	4.9733×10^{-3}	1.1418×10^{-3}	0.954	6.3509×10^{-3}	8.8842×10^{-3}	0.136	1.3391×10^{-3}	1.3007×10^{-3}	0.792
Sick above 3 days	1.3576×10^{-3}	6.3373×10^{-3}	0.047	5.4398×10^{-3}	1.0328×10^{-2}	0.059	1.3853×10^{-3}	1.2533×10^{-3}	0.897
Preadmission seizures	1.3164×10^{-3}	9.3656×10^{-3}	0.013	6.8099×10^{-3}	7.65899×10^{-3}	0.604	1.6374×10^{-3}	1.2044×10^{-3}	0.194
Preadmission antibiotics	2.3151×10^{-3}	1.8842×10^{-3}	0.880	6.7108×10^{-3}	7.0787×10^{-3}	0.974	1.4770×10^{-3}	1.2961×10^{-3}	0.796
Seizures at admission	1.1140×10^{-3}	8.1868×10^{-3}	0.001	6.8992×10^{-3}	7.8746×10^{-3}	0.729	1.6242×10^{-3}	1.2570×10^{-3}	0.494

[a]Median cerebrospinal fluid concentrations in nmol/L

Table S3. Supplemental digital content**.** Associations between admission cerebrospinal fluid protein oxidation biomarker ratios of o-Tyr-Phe, 3Cl-Tyr/p-Tyr and $3NO_2$-Tyr/p-Tyr (median) and course of illness and patient outcome, calculated using Mann-Whitney U-test.

*Any hearing impairment = Does not hear 40dB either ear

**Characteristic**	o-Tyr/Phe			3Cl-Tyr/p-Tyr			$3NO_2$-Tyr/p-Tyr		
	Yes	No	*p* value	Yes	No	*p* value	Yes	No	*p* value
Seizures at the ward	1.3682×10^{-3}	4.5530×10^{-3}	0.081	5.9915×10^{-3}	8.3584×10^{-3}	0.719	1.3480×10^{-3}	1.2497×10^{-3}	0.873
Seizures during hospital stay (on admission/at the ward)	1.3807×10^{-3}	8.1868×10^{-3}	0.022	6.760×10^{-3}	1.0296×10^{-2}	0.749	1.2570×10^{-3}	1.3436×10^{-3}	0.885
Focal seizures during illness	1.290×10^{-3}	4.9984×10^{-3}	0.039	6.7560×10^{-3}	7.0787×10^{-3}	0.726	1.2962×10^{-3}	1.3023×10^{-3}	0.992
Secondary fever	1.190×10^{-3}	7.2621×10^{-3}	0.009	5.43981×10^{-3}	7.8467×10^{-3}	0.958	$1.53671.3025\times10^{-3}$	1.3025×10^{-3}	0.199
Glasgow Outcome Score below optimal									
On Day 7 of treatment	1.3164×10^{-3}	6.3373×10^{-3}	0.031	7.1594×10^{-3}	6.7101×10^{-3}	0.426	1.3481×10^{-3}	1.4225×10^{-3}	0.948

**Mild hearing impairment = Does not hear 40dB

One month after discharge	1.2637×10^{-3}	8.1868×10^{-3}	0.004	6.99799×10^{-3}	7.8746×10^{-3}	0.970	1.3391×10^{-3}	1.6528×10^{-3}	0.698
Death									
Within 24h of treatment	1.5108×10^{-3}	8.7040×10^{-3}	0.756	1.2029×10^{-2}	6.3508×10^{-3}	0.241	1.2533×10^{-3}	1.3436×10^{-3}	1.00
By Day 7 of treatment	1.2460×10^{-3}	2.7687×10^{-3}	0.398	7.0787×10^{-3}	6.7101×10^{-3}	0.624	1.2044×10^{-3}	1.4225×10^{-3}	0.332
Severe neurological sequelae									
On Day 7 (n = 48)	1.3769×10^{-3}	1.3682×10^{-3}	0.283	8.3495×10^{-3}	6.9980×10^{-3}	0.356	1.8997×10^{-3}	1.4497×10^{-3}	0.653
One month after discharge (n = 31)	1.3164×10^{-3}	3.7323×10^{-3}	0.285	7.6310×10^{-3}	5.9334×10^{-3}	0.422	2.1466×10^{-3}	2.1466×10^{-3}	0.285
Any neurological sequelae									
On Day 7 (n = 51)	1.2272×10^{-3}	1.27198×10^{-2}	0.0002	4.9456×10^{-3}	1.1382×10^{-2}	0.159	1.6673×10^{-3}	1.2008×10^{-3}	0.128
One month after discharge (n = 31)	1.2097×10^{-3}	9.3656×10^{-3}	0.03	4.9447×10^{-3}	1.1397×10^{-2}	0.029	1.9297×10^{-3}	1.4787×10^{-3}	0.381
Ataxia									
On Day 7 of treatment (n = 51)	1.3423×10^{-3}	1.27198×10^{-2}	0.0002	5.4398×10^{-3}	1.3451×10^{-3}	0.159	1.6601×10^{-3}	1.2242×10^{-3}	0.128

One month after discharge (n = 31)	1.2630×10^{-3}	9.3656×10^{-3}	0.015	4.9451×10^{-3}	8.2191×10^{-3}	0.087	1.7034×10^{-3}	1.6272×10^{-3}	0.603
Hearing impairment Day 7 (n = 45)									
Any hearing impairment	1.3682×10^{-3}	6.6052×10^{-3}	0.106	6.8099×10^{-3}	4.5121×10^{-3}	0.601	1.6528×10^{-3}	1.1935×10^{-3}	0.133
Mild impairment*	1.4373×10^{-3}	2.8402×10^{-3}	0.903	7.8761×10^{-3}	4.93203×10^{-3}	0.207	1.9297×10^{-3}	1.1082×10^{-3}	0.035
Moderate impairment**	1.2634×10^{-2}	2.3896×10^{-3}	0.780	6.750×10^{-3}	6.750×10^{-3}	0.747	6.750×10^{-4}	1.3853×10^{-3}	0.090
Severe impairment***	1.3422×10^{-3}	6.750×10^{-3}	0.298	6.1945×10^{-3}	6.7301×10^{-3}	0.673	1.3341×10^{-3}	1.2570×10^{-3}	0.327
Hearing impairment at one month									
Any hearing impairment (n = 27)	1.3423×10^{-3}	5.0235×10^{-3}	0.200	8.3775×10^{-3}	4.9756×10^{-3}	0.300	1.7657×10^{-3}	1.3482×10^{-3}	0.373
Mild impairment	1.7147×10^{-3}	4.9734×10^{-3}	0.279	5.4339×10^{-3}	7.4434×10^{-3}	0.570	6.5489×10^{-3}	6.5489×10^{-3}	0.051
Moderate impairment	1.3682×10^{-3}	4.7631×10^{-3}	0.856	1.2437×10^{-2}	7.0767×10^{-3}	0.532	1.6016×10^{-3}	1.4126×10^{-3}	0.914

Severe impairment^	9.8793×10^{-4}	4.9984×10^{-3}	0.021	7.4433×10^{-3}	7.2923×10^{-3}	0.532	1.0346×10^{-3}	1.5393×10^{-3}	0.971

***Moderate hearing impairment = Does not hear 60dB

****Severe hearing impairment = Does not hear 80dB

^Severe hearing impairment n = 3, No severe hearing impairment n = 24
